# Correlation of ^68^Ga-DOTATATE and ^18^F-FDG PET/CT Uptake Parameters with PASS and GAPP Scores in Pheochromocytoma

**DOI:** 10.3390/diagnostics16172858

**Published:** 2026-09-05

**Authors:** Ilknur Ozturk Unsal, Enes Ucgul, Ceren Karacalik Unver, Nur Dizdar, Sema Hepsen, Murat Calapkulu, Aysenur Erdem Karaoglu, Derya Cayir, Erman Cakal

**Affiliations:** 1 Department of Endocrinology and Metabolism, University of Health Sciences, Etlik City Hospital, 06170 Ankara, Turkey; enes-ucgul@hotmail.com (E.U.); cerenkaracalik@gmail.com (C.K.U.); semahepsen@gmail.com (S.H.); calapkulumurat89@gmail.com (M.C.); ermancakal@hotmail.com (E.C.); 2 Department of Nuclear Medicine, University of Health Sciences, Etlik City Hospital, 06170 Ankara, Turkey; fnuraydinbelge@gmail.com (N.D.); aysenurerdemmd@gmail.com (A.E.K.); drderyaors@gmail.com (D.C.)

**Keywords:** pheochromocytoma, ^68^Ga-DOTATATE PET/CT, ^18^F-FDG PET/CT, PASS, GAPP score

## Abstract

**Background:** Pheochromocytomas and paragangliomas (PPGLs) are rare neuroendocrine tumors with highly variable biological behavior. Somatostatin receptor (SSTR)-based imaging with ^68^Ga-DOTATATE PET/CT has become an important functional imaging modality for evaluating these tumors. In contrast, ^18^F-FDG PET/CT reflects tumor glucose metabolism and has been associated with more aggressive tumor phenotypes. This study aimed to evaluate associations between ^68^Ga-DOTATATE and ^18^F-FDG PET/CT-derived parameters and postoperative PASS and GAPP scores in patients with pheochromocytoma. **Materials and Methods:** Thirty patients with histologically confirmed pheochromocytoma who underwent ^68^Ga-DOTATATE PET/CT were retrospectively reviewed. Among them, 11 patients also underwent ^18^F-FDG PET/CT and were included in the comparative imaging analysis. **Results:** Tumor size showed strong positive correlations with both SSTR-TV (ρ = 0.725, *p* < 0.001) and SSTR-TL (ρ = 0.755, *p* < 0.001). ^18^F-FDG SUVmax was positively correlated with PASS (ρ = 0.616, *p* = 0.044) and GAPP scores (ρ = 0.720, *p* = 0.012). ^18^F-to-^68^Ga SUVmax ratio showed significant positive correlations with the PASS (ρ = 0.737, *p* = 0.010) and the GAPP score (ρ = 0.661, *p* = 0.027). **Conclusions:** In this exploratory study, ^68^Ga-derived volumetric parameters were primarily associated with tumor size, whereas ^18^F SUVmax and the ^18^F/^68^Ga SUVmax ratio were associated with PASS and GAPP scores in the small dual-tracer subgroup. These findings require validation in larger cohorts.

## 1. Introduction

Pheochromocytomas and paragangliomas (PPGLs) are rare catecholamine-producing neuroendocrine tumors derived from chromaffin cells of adrenal or extra-adrenal origin. Most PPGLs (80–85%) arise in the adrenal medulla, while the remaining 15–20% originate from extra-adrenal chromaffin tissue [1]. Although uncommon, with an estimated incidence of 0.04–0.95 per 100,000, PPGLs are clinically important [2] because of their catecholamine secretion, metastatic potential, and risk of severe cardiovascular complication [3]. The classical presentation includes episodic headache, palpitations, diaphoresis, and hypertension; however, clinical manifestations are heterogeneous, and up to one-third of patients may be asymptomatic at diagnosis [3,4].

Pheochromocytomas account for approximately 4–7% of adrenal incidentalomas, and biochemical screening is recommended for patients with young-onset or resistant hypertension, symptoms of catecholamine excess, adrenal incidentalomas with unenhanced CT attenuation > 10 Hounsfield units (HU), or catecholamine-related cardiac manifestations [5,6,7]. Plasma free metanephrines and urinary fractionated metanephrines are the preferred diagnostic tests, outperforming catecholamine measurements; plasma free metanephrines demonstrated 97.9% sensitivity and 94.2% specificity in a large multicenter prospective study [8]. Anatomical assessment of PPGLs is primarily performed using computed tomography (CT) and magnetic resonance imaging, while functional imaging is used for lesion characterization, staging, and detection of multifocal or metastatic disease. PET/CT with ^18^F-FDG or ^68^Ga-DOTA-coupled somatostatin receptor ligands offers superior sensitivity and spatial resolution compared with conventional scintigraphy, with ^68^Ga-DOTA-peptide imaging being particularly valuable in PPGLs because of frequent overexpression of somatostatin receptor subtype 2 [9,10].

Risk stratification in pheochromocytoma remains clinically important because all PPGLs have metastatic potential. Histopathological scoring systems, including the Pheochromocytoma of the Adrenal Gland Scaled Score (PASS) and the Grading System for Adrenal Pheochromocytoma and Paraganglioma (GAPP), are used to assess aggressive biological behavior, with higher scores associated with adverse clinical outcomes [11,12]. Although ^68^Ga-DOTATATE PET/CT (^68^Ga) and ^18^F-FDG PET/CT (^18^F) provide complementary biological information by reflecting somatostatin receptor expression and glucose metabolic activity, respectively, evidence linking these imaging-derived parameters to PASS and GAPP scores remains limited [13]. In particular, whether ^18^F and ^68^Ga SUVmax values, or their ratio, may serve as non-invasive imaging markers of histopathological aggressiveness has not been systematically evaluated.

This study aimed to evaluate associations between ^68^Ga and ^18^F-derived imaging parameters and biochemical activity, PASS, and GAPP scores in patients with histologically confirmed pheochromocytoma. We also explored whether the ^18^F-to-^68^Ga SUVmax ratio was associated with higher histopathological scores and could discriminate between predefined PASS and GAPP categories.

## 2. Materials and Methods

### 2.1. Study Design and Patient Population

**Figure 1:**
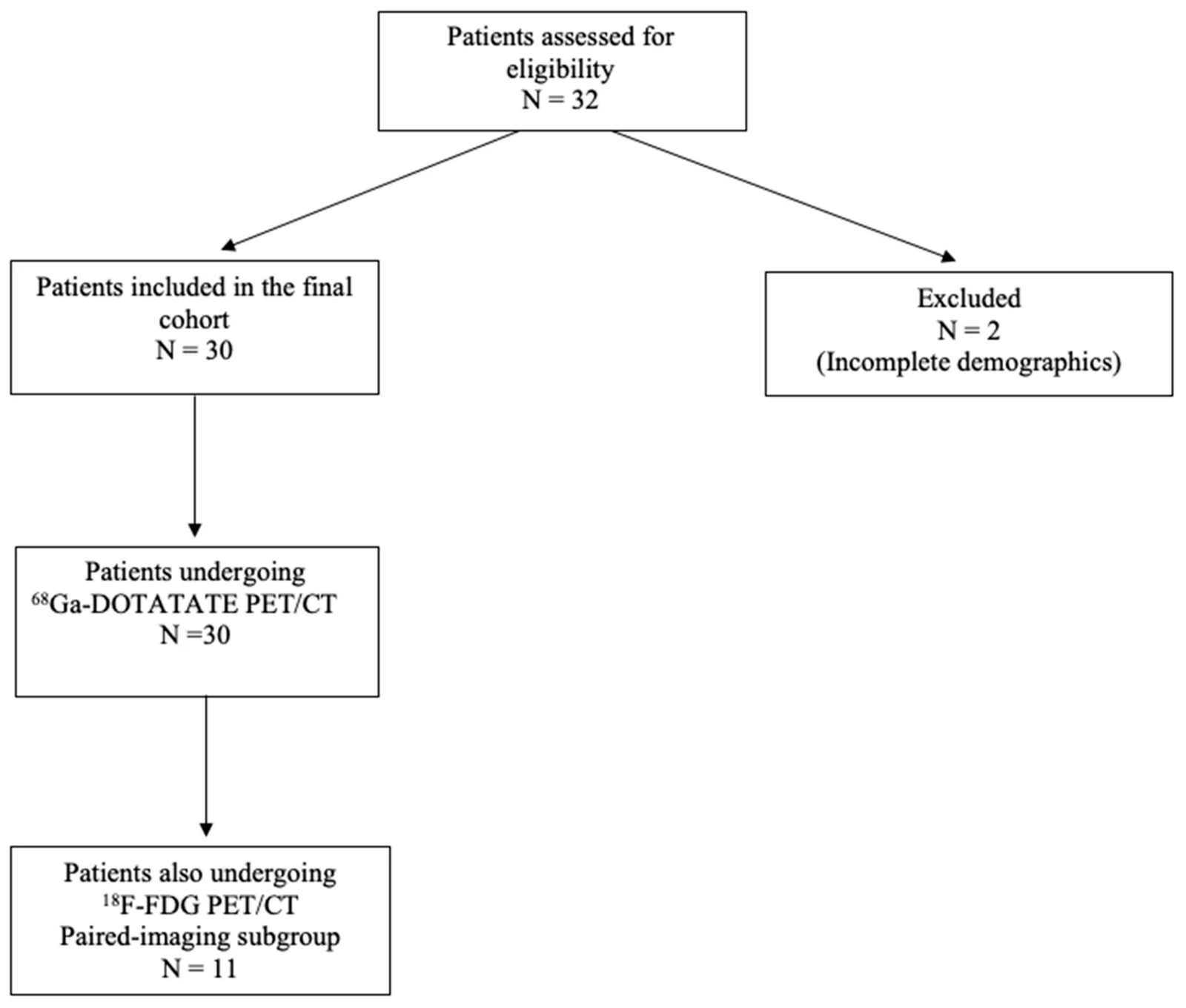
Flowchart of patient selection and imaging protocol.

This retrospective single-center study was conducted at Etlik City Hospital. Medical records of patients with histopathologically confirmed pheochromocytoma who underwent adrenalectomy between October 2022 and December 2025 were reviewed. A total of 32 patients were initially screened for eligibility. Two patients were excluded due to incomplete demographic and clinical data, resulting in a final study cohort of 30 patients (Figure 1). Demographic characteristics, clinical presentation, biochemical findings, radiological features, histopathological parameters, and PET/CT imaging data were retrospectively retrieved from the institutional electronic medical record system and analyzed.

The study protocol was approved by the Institutional Ethics Committee of Etlik City Hospital (Approval No: AEŞH-BADEK2-2025-320) and was conducted in accordance with the ethical standards of the Declaration of Helsinki.

### 2.2. Clinical, Biochemical, and Histopathological Assessment

Demographic and clinical data, including age, sex, presenting symptoms, blood pressure status, tumor size, and radiological findings, were collected from medical records. Blood pressure categories were classified according to contemporary hypertension guidelines [14]. Biochemical evaluation included measurements of plasma free metanephrine and normetanephrine as well as 24 h urinary metanephrine and normetanephrine levels. Plasma free metanephrine and normetanephrine concentrations were measured using liquid chromatography–tandem mass spectrometry (LC-MS/MS).

PASS and GAPP scores were obtained from the original postoperative pathology reports during the retrospective review of archived medical records. Because of the retrospective design, we could not reliably determine the exact number of reporting pathologists. Pathologists experienced in adrenal pathology issued the original reports, and no centralized histopathological re-review was performed. We performed histopathological risk stratification using both PASS and GAPP. For categorical analyses, a PASS of ≥4 was defined as high risk, whereas a PASS of <4 was considered low risk. Similarly, GAPP scores were dichotomized according to the original grading categories: tumors with a GAPP score ≥ 3 were classified as moderate-to-high histopathological risk, while those with a GAPP score of 0–2 were classified as low risk [11,12].

### 2.3. ^68^Ga and ^18^F Imaging and Quantitative Analysis

All PET/CT examinations were performed using an integrated six-slice Discovery IQ PET/CT system (GE HealthCare, Milwaukee, WI, USA). For ^18^F imaging, patients fasted for at least 6 h prior to tracer administration, and blood glucose levels were confirmed to be below 180 mg/dL before imaging. Approximately 3.5 MBq/kg of ^18^F was administered intravenously, and image acquisition was performed approximately 60 min after tracer injection. For ^68^Ga imaging, patients received an intravenous injection of ^68^Ga at 185–200 MBq according to established procedural guidelines [15]. PET/CT acquisition was performed 45–60 min after tracer administration. For both imaging modalities, low-dose CT images (120 kV, 10–90 mA, 3 mm slice thickness) were acquired for attenuation correction and anatomical localization.

An experienced nuclear medicine physician reviewed all PET/CT images using a dedicated workstation. The image analysis was performed independently of the pathological findings, and the nuclear medicine physician was blinded to the histopathological results during image evaluation. For quantitative analysis, semi-automatic volumes of interest were drawn around the primary tumor. Physiological tracer uptake and adjacent organs with high physiological activity were carefully excluded from the tumor volume. When necessary, the contours were manually adjusted to prevent the inclusion of adjacent normal structures, physiological uptake, or areas of activity unrelated to the primary tumoral lesion. For ^18^F, the following semiquantitative parameters were calculated: maximum standardized uptake value (SUVmax), mean standardized uptake value (SUVmean), metabolic tumor volume (MTV), and total lesion glycolysis (TLG). MTV was calculated using a threshold corresponding to 42% of SUVmax, and TLG was calculated as the product of MTV and SUVmean. For ^68^Ga, SUVmax, SUVmean, somatostatin receptor tumor volume (SSTR-TV), and somatostatin receptor total lesion activity (SSTR-TL) were calculated. SSTR-TV was determined using a threshold of 42% of SUVmax, and SSTR-TL was calculated as the product of SUVmean and SSTR-TV obtained using the same threshold [16,17,18,19]. For patients who underwent both ^18^F and ^68^Ga examinations, ^18^F-to-^68^Ga SUVmax ratios were also calculated. We performed the two PET/CT scans 3–5 days apart, with no treatment administered between examinations. We obtained both SUVmax measurements from the same primary tumor; we did not include metastatic lesions.

### 2.4. Statistical Analysis

We performed statistical analyses using IBM SPSS Statistics version 27. We evaluated the distribution of continuous variables using the Shapiro–Wilk test and visual assessment of histograms and Q–Q plots. Normally distributed continuous variables are presented as mean ± standard deviation (SD), whereas non-normally distributed variables are expressed as median and interquartile range (IQR). Categorical variables are presented as frequencies and percentages. Associations between biochemical, pathological, radiological, and PET/CT-derived parameters were assessed using Spearman’s rank correlation coefficient (ρ). To evaluate whether associations between SSTR-derived volumetric parameters and plasma normetanephrine were independent of tumor size, we performed partial Spearman correlation analyses while controlling for tumor size. For this analysis, we rank-transformed the variables and calculated partial correlations between the ranked variables, entering tumor size as the control variable. The primary analyses included correlations between ^68^Ga SUVmax, ^18^F SUVmax, and the ^18^F/^68^Ga SUVmax ratio and PASS and GAPP, as these analyses directly addressed the central study hypothesis. We considered all remaining correlations between imaging, biochemical, and morphological parameters as secondary exploratory analyses. We did not apply multiplicity adjustment to these exploratory analyses; therefore, we interpreted their nominal *p* values as hypothesis-generating rather than confirmatory. We also performed receiver operating characteristic (ROC) curve analysis to evaluate the discriminatory performance of the ^18^FDG/^68^Ga SUVmax ratio in predicting predefined histopathological risk categories. PASS and GAPP scores were dichotomized using established thresholds: PASS ≥ 4 was defined as high risk, and GAPP ≥ 3 as moderate-to-high risk [10,11]. The area under the curve (AUC), standard error, 95% confidence interval (CI), and *p*-value were calculated using a nonparametric method. The optimal cut-off for the ^18^F-FDG/^68^Ga SUVmax ratio was determined using the Youden index, calculated as sensitivity + specificity − 1. Sensitivity and specificity at the optimal cut-off were reported. All statistical tests were two-tailed, and a *p*-value < 0.05 was considered statistically significant.

## 3. Results

A total of 30 patients with histologically confirmed pheochromocytoma were included in the study. The mean age at diagnosis was 53.8 ± 14.8 years, and the patients were predominantly female, with 19 women (63.3%) and 11 men (36.7%). Adrenal incidentaloma was the most common presentation, accounting for 60.0% of cases (*n* = 18), whereas uncontrolled hypertension was the presenting feature in 23.3% (*n* = 7). Pheochromocytoma-related symptoms were reported in four patients (13.3%), while one patient (3.3%) was diagnosed in the context of a hereditary syndrome. At the time of diagnosis, hypertension was present in 20 patients (66.7%). Stage 1 hypertension was observed in 43.3% of patients (*n* = 13), followed by stage 2 hypertension in 23.3% (*n* = 7). The percentages of patients with elevated and normal blood pressure were 20.0% (*n* = 6) and 13.3% (*n* = 4), respectively. Tumors had a median diameter of 4.15 cm (IQR, 2.5–5.5) and a median attenuation of 37.5 Hounsfield units (IQR, 34.5–48.2) on CT. Biochemically, patients showed markedly elevated catecholamine metabolite levels, with median plasma normetanephrine, plasma metanephrine, 24 h urinary normetanephrine, and 24 h urinary metanephrine concentrations of 1409 pg/mL (IQR, 551–3812), 475 pg/mL (IQR, 300–675), 2933 µg/day (IQR, 692–5312), and 698 µg/day (IQR, 166–2391), respectively. The median Ki-67 proliferation index was 2% (IQR, 1–3). Two patients (6.7%) had metastatic disease at diagnosis. Also, the mean ^68^Ga SUVmax and SUVmean values were 22.3 ± 2.08 and 13.18 ± 6.07, respectively, whereas the mean SSTR-TV and SSTR-TL values were 25.02 ± 5.6 and 327 ± 73.1. Among the 11 patients who underwent ^18^F imaging, the mean SUVmax and SUVmean values were 4.44 ± 2.38 and 2.64 ± 1.45, respectively, and the mean MTV and TLG values were 16.6 ± 9.54 and 58.4 ± 40.8, respectively. Histopathological risk assessment showed a median GAPP score of 4.5 (2–6) and a median PASS of 5.0 (2–6). Detailed baseline demographic, clinical, biochemical, and tumor characteristics are summarized in Table 1.

As noted in Table 1, we identified metastatic disease at diagnosis in two patients, whose clinical and histopathological characteristics are described below. The first was a 22-year-old woman who presented with symptoms suggestive of pheochromocytoma. Her plasma metanephrine and normetanephrine concentrations were fourfold and 15-fold above the upper limits of normal, respectively. Imaging revealed a 2.5 cm adrenal tumor with metastases to three para-aortic lymph nodes. The adrenal lesion had SUVmax values of 12.7 on ^68^Ga and 6.0 on ^18^F. Histopathological evaluation showed a GAPP score of 5 and a PASS of 3. The second patient was a 71-year-old woman evaluated for iron-deficiency anemia, during which a sigmoid colonic mass was detected. Histopathological and immunohistochemical comparison supported the colonic lesion as a metastasis from the adrenal pheochromocytoma. Further imaging revealed a 2 cm right adrenal lesion, with SUVmax values of 33.2 on ^68^Ga and 6.64 on ^18^F. Plasma catecholamine metabolite concentrations were approximately fourfold above the upper limit of normal. Histopathological examination of the adrenal tumor confirmed pheochromocytoma, with GAPP and PASS of 4 and 4, respectively.

As shown in Table 2, 20 patients (66.7%) in the overall cohort had PASS ≥ 4, and 20 (66.7%) had GAPP scores ≥ 3. Similar proportions were observed in the dual-tracer subgroup, with seven patients (63.6%) in each higher-score category.

Following this descriptive characterization, we assessed catecholamine metabolites and PET/CT characteristics using correlation analysis. Tumor size and plasma normetanephrine levels were positively correlated with both SSTR-TV (ρ = 0.725, *p* < 0.001 and ρ = 0.532, *p* = 0.002, respectively) and SSTR-TL (ρ = 0.755, *p* < 0.001 and ρ = 0.486, *p* = 0.006, respectively). After adjustment for tumor size using partial Spearman correlation, neither SSTR-TV nor SSTR-TL was significantly associated with plasma normetanephrine (SSTR-TV: partial ρ = 0.218, *p* = 0.256; SSTR-TL: partial ρ = 0.112, *p* = 0.560). In contrast, tumor size was positively correlated with plasma normetanephrine (ρ = 0.563, *p* = 0.001), suggesting that the observed imaging–biochemical relationships may reflect tumor burden. Furthermore, we found no significant associations between plasma normetanephrine levels and ^18^F-derived volumetric parameters. No significant correlations were found between tumor size and ^18^F-derived volumetric indices (Table 3).

In the subgroup of 11 patients who underwent ^18^F, SUVmax was positively correlated with both PASS (ρ = 0.616, *p* = 0.044) and GAPP scores (ρ = 0.720, *p* = 0.012). In the overall cohort of 30 patients, ^68^Ga SUVmax was not significantly correlated with either PASS (ρ = 0.162, *p* = 0.392) or GAPP scores (ρ = 0.062, *p* = 0.745) (Table 4). Notably, the ^18^F-to-^68^Ga SUVmax ratio showed significant positive correlations with the PASS (ρ = 0.737, *p* = 0.010) and the GAPP score (ρ = 0.661, *p* = 0.027) (Table 5).

**Figure 2:**
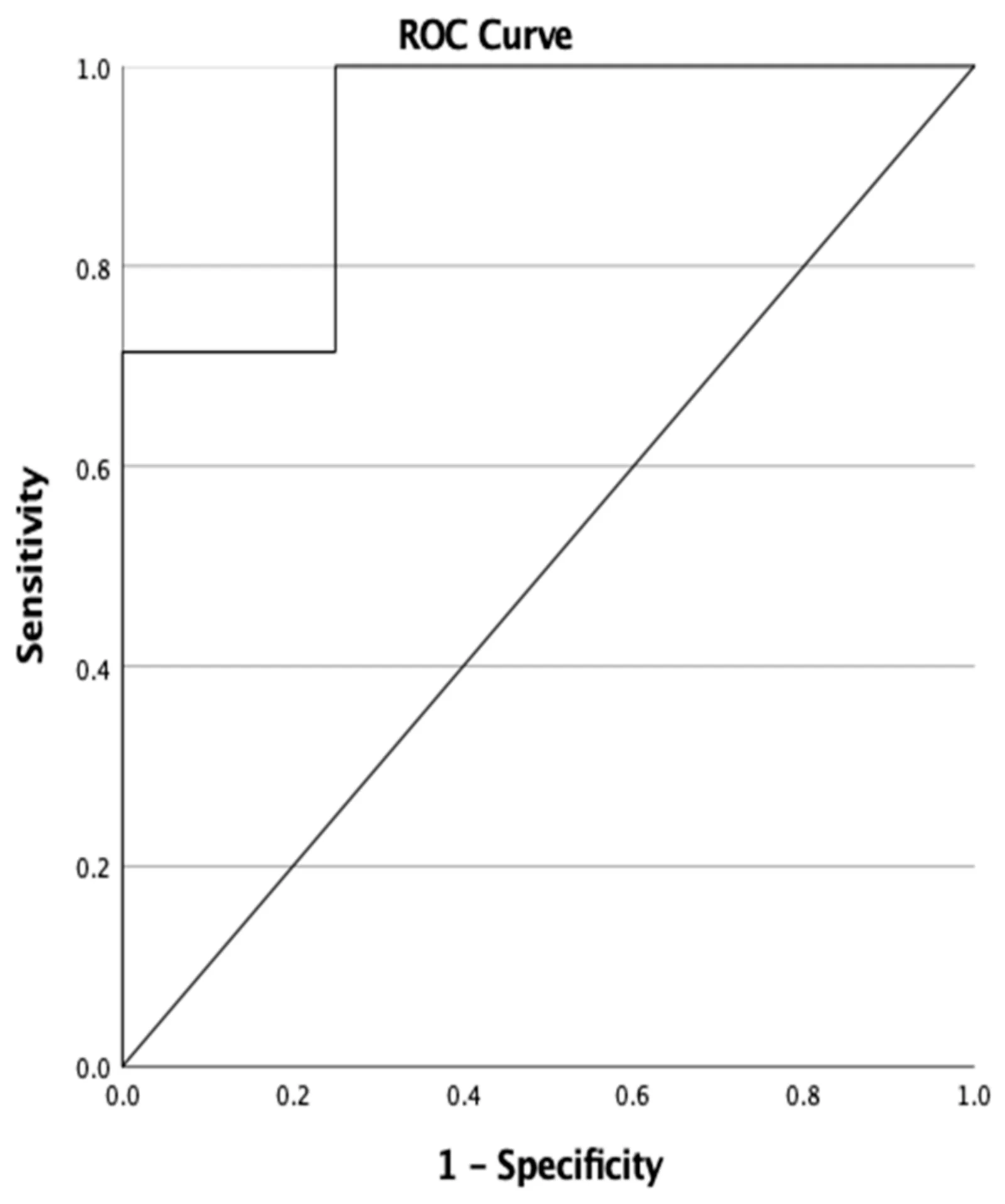
ROC curve analysis of the ^18^F/^68^Ga SUVmax ratio for identifying patients with a high PASS.

**Figure 3:**
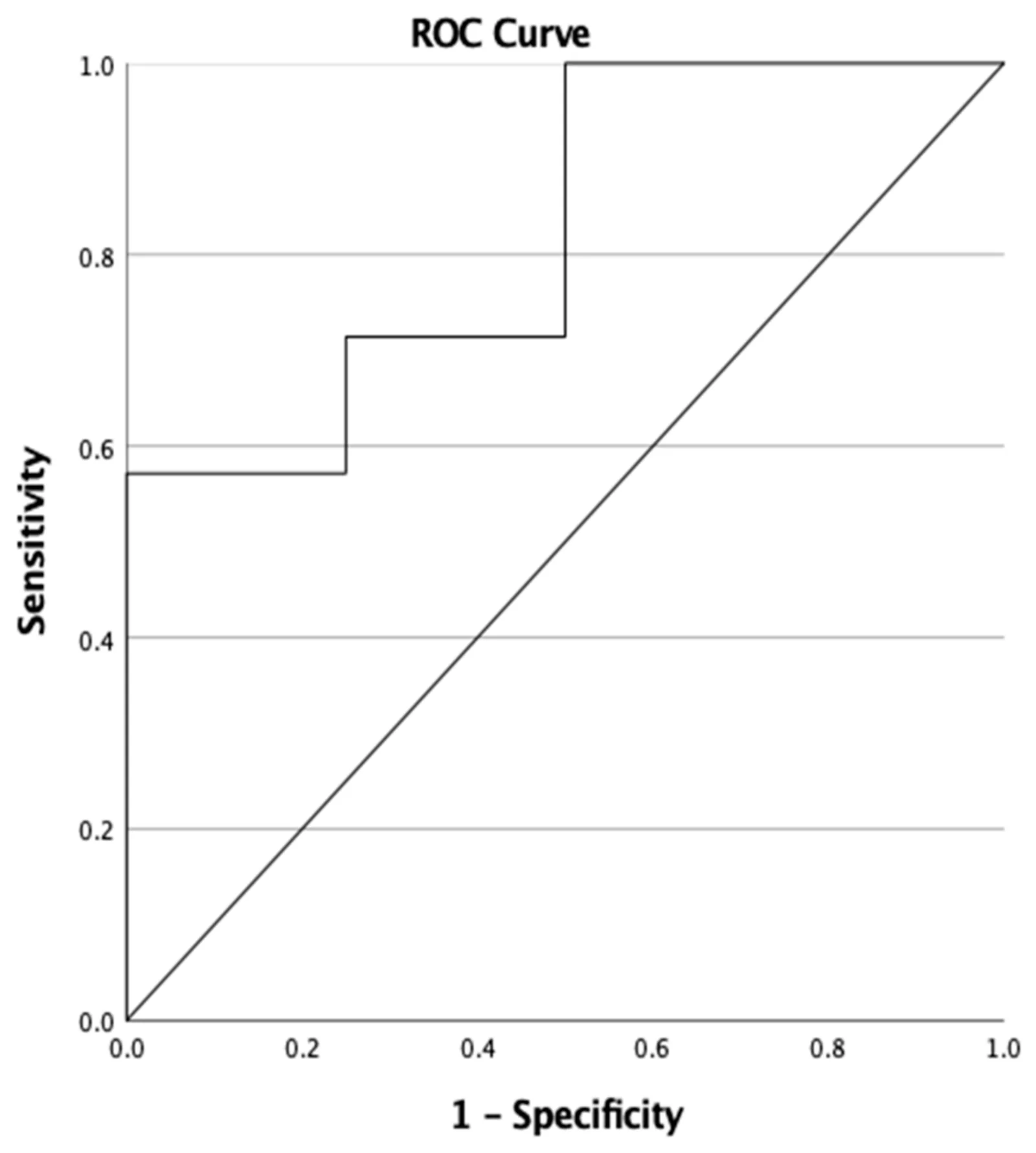
ROC curve of the ^18^F/^68^Ga SUVmax ratio for identifying patients with a moderate-to-high GAPP score.

We conducted exploratory ROC curve analyses to assess the discriminatory performance of the ^18^F/^68^Ga SUVmax ratio for predefined PASS and GAPP categories. As shown in Figure 2, the ^18^F/^68^Ga SUVmax ratio demonstrated an AUC of 0.929 (95% CI, 0.764–1.000; *p* = 0.023) for discriminating patients with PASS ≥ 4 from those with PASS < 4. The sample-derived Youden-derived cutoff of 0.156 yielded an apparent sensitivity of 100% and specificity of 75%. For discriminating patients in the moderate-to-high GAPP (GAPP ≥ 3) from those in the low GAPP (GAPP 0–2), the ratio demonstrated an AUC of 0.821 (95% CI, 0.560–1.000; *p* = 0.089) (Figure 3). The corresponding sample-derived cutoff was 0.242, with 57.1% sensitivity and 100% specificity. Also, Table 6 presents the corresponding 2 × 2 distributions.

## 4. Discussion

In this retrospective study, ^68^Ga-derived volumetric parameters were strongly associated with tumor size. They were also associated with plasma normetanephrine levels in unadjusted analyses; however, only the imaging–normetanephrine associations became nonsignificant after adjustment for tumor size. In the smaller subgroup that underwent dual-tracer imaging, ^18^F SUVmax and the ^18^F-to-^68^Ga SUVmax ratio were positively correlated with PASS and GAPP scores. These findings may suggest that receptor-based volumetric and glucose-metabolic imaging parameters provide different, potentially complementary aspects of pheochromocytoma. However, because the analyses were cross-sectional, based on histopathological surrogate scores, and limited by a small dual-tracer subgroup, they should be interpreted as hypothesis-generating rather than evidence of prognostic or clinical utility.

In the present study, ^68^Ga-derived volumetric parameters were positively associated with tumor size and plasma normetanephrine concentrations in unadjusted analyses. However, after adjustment for tumor size, the associations of SSTR-TV and SSTR-TL with plasma normetanephrine were attenuated and were no longer statistically significant. These findings suggest that tumor burden largely explained the observed unadjusted imaging–biochemical associations, rather than an independent relationship between SSTR-derived volumetric parameters and catecholamine secretion. Consistent with our unadjusted findings, a recent PPGL study using a different SSTR-targeting radioligand, ^18^F-SiTATE, also reported correlations between SSTR-PET volumetric parameters and plasma normetanephrine; however, that analysis did not account for tumor size [20]. Because plasma normetanephrine levels increase with tumor size, the loss of these correlations after adjustment in our study suggests they were largely driven by tumor burden. Thus, we could not demonstrate that SSTR-TV or SSTR-TL provides information on catecholamine secretion beyond what tumor size conveys [21].

The literature supports the prognostic and biological relevance of volumetric SSTR-PET parameters in neuroendocrine neoplasms [22]. A recent meta-analysis demonstrated that patients with higher SSTR-derived tumor volumes have approximately a two-fold increased risk of disease progression and poorer survival outcomes. These observations reflect that volumetric PET metrics integrate both tumor burden and receptor expression, thereby reflecting tumor biology more accurately than conventional uptake-based parameters alone [21]. In addition, previous studies have shown that volumetric analysis may improve lesion characterization beyond standard imaging measurements. For example, Denizmen et al. reported that ^68^Ga-derived volumetric parameters, particularly SSTR-TV, provided high diagnostic accuracy in differentiating pheochromocytomas from adrenal adenomas [23]. In the overall cohort, ^68^Ga SUVmax was not significantly associated with either PASS or GAPP scores. In contrast, ^18^F SUVmax was positively correlated with both histopathological scores in the smaller dual-tracer subgroup. These findings suggest that SSTR-targeted PET/CT may primarily reflect tumor burden rather than pathological aggressiveness. Collectively, our results suggest that ^68^Ga-derived volumetric parameters may reflect tumor burden; however, the present analysis did not demonstrate incremental biochemical information beyond that provided by tumor size.

In contrast to ^68^Ga-derived volumetric parameters, ^18^F uptake was more closely associated with histopathological indicators of tumor aggressiveness. In our cohort, ^18^F SUVmax showed significant positive correlations with both PASS and GAPP scores, whereas ^68^Ga SUVmax showed no associations. These findings suggest that increased glucose metabolism may more accurately reflect biological aggressiveness than somatostatin receptor expression in pheochromocytoma. In neuroendocrine neoplasms, tumor differentiation is closely linked to imaging phenotype. Well-differentiated tumors typically maintain high somatostatin receptor expression and demonstrate intense uptake on SSTR-targeted imaging, whereas poorly differentiated tumors are characterized by increased glycolytic activity and consequently higher ^18^F uptake. Several studies evaluating dual-tracer PET imaging have described this biological shift [24,25,26,27]. Kayani et al. reported that low-grade neuroendocrine tumors accumulated more ^68^Ga than ^18^F. By contrast, higher-grade lesions demonstrated progressively increased FDG avidity [21]. Combining the two tracers may provide complementary biological information by integrating receptor expression and glucose metabolism. Similarly, studies in pheochromocytoma and paraganglioma have shown significantly higher ^18^F uptake in tumors with aggressive clinical behavior compared with those with less aggressive biological behavior [28].

An important finding of our study was the strong correlation between the ^18^F-to-^68^Ga SUVmax ratio and both PASS and GAPP scores. Furthermore, the ratio showed high apparent discriminatory performance for identifying patients with higher PASS in this small exploratory subgroup. Rather than evaluating glucose metabolism or receptor expression alone, this parameter combines both biological processes into a single metric. Higher ^18^F-to-^68^Ga SUVmax ratios may therefore reflect a relative increase in glucose metabolism, accompanied by reduced somatostatin receptor expression, suggesting a transition from a well-differentiated, receptor-dominant phenotype toward a more aggressive, dedifferentiated tumor biology [26,27]. These findings support further evaluation of the exploratory associations between dual-tracer PET/CT parameters and predefined histopathological score categories in pheochromocytoma. Nevertheless, given the limited number of patients who underwent dual-tracer imaging, larger prospective studies are required to validate the clinical utility of this imaging biomarker.

This study has several limitations. Its retrospective, single-center design and small sample size limit the precision and generalizability of the findings. We performed ^18^F only in a small subgroup without a prespecified selection protocol, introducing potential selection and spectrum bias. The ROC analyses were based on very small groups; therefore, the thresholds identified should be regarded solely as exploratory, sample-derived values rather than proposed or clinically applicable cutoffs. Moreover, we derived and evaluated these thresholds in the same dataset without internal or external validation. Consequently, the reported AUC, sensitivity, and specificity may overestimate the true discriminatory performance. We evaluated PASS and GAPP as surrogate histopathological risk measures rather than direct clinical outcomes, and they do not perfectly predict metastatic behavior. The absence of longitudinal follow-up precluded assessment of recurrence, progression, metastasis-free survival, or disease-specific survival. We did not systematically incorporate genetic background, although PPGL genotype may influence functional imaging phenotype. In addition, we tested multiple exploratory correlations without adjustment for multiple comparisons, increasing the risk of type I error.

## 5. Conclusions

In conclusion, this exploratory study suggests that dual-tracer PET/CT may provide complementary information on the biological characteristics of pheochromocytoma. ^68^Ga-derived volumetric parameters were associated with tumor size, whereas ^18^F and the ^18^F/^68^Ga SUVmax ratio were associated with PASS and GAPP scores. These cross-sectional associations should be considered hypothesis-generating and do not establish the prognostic or risk-stratification utility of dual-tracer imaging. Independent validation in larger, prospective multicenter cohorts, incorporating molecular genotype and longitudinal clinical outcomes, is required to determine whether these imaging parameters have clinically meaningful prognostic value.

## Figures and Tables

**Table 1:** Baseline demographic, clinical, biochemical, and tumor characteristics of patients with pheochromocytoma.

Variable	Value
**Age, years**	53.8 ± 14.8
**Sex, *n* (%)**	
Female	19 (63.3)
Male	11 (36.7)
**Reason for presentation**	
Adrenal incidentaloma	18 (60.0)
Uncontrolled hypertension	7 (23.3)
Syndromic presentation	1 (3.3)
Pheochromocytoma-related symptoms	4 (13.3)
**Blood pressure category, *n* (%)**	
Normal blood pressure	4 (13.3)
Elevated blood pressure	6 (20.0)
Stage 1 hypertension	13 (43.3)
Stage 2 hypertension	7 (23.3)
**Tumor characteristics**	
Tumor size, cm	4.15 (2.5–5.5)
Tumor attenuation on CT, HU	37.5 (34.5–48.2)
Ki-67 index, %	2 (1–3)
Metastatic disease at diagnosis, *n* (%)	2 (6.7)
**Biochemical parameters**	
Plasma normetanephrine, pg/mL	1409 (551–3812)
Plasma metanephrine, pg/mL	475 (300–675)
24 h urinary normetanephrine, µg/24 h	2933 (692–5312)
24 h urinary metanephrine, µg/24 h	698 (166–2391)
**^68^Ga-DOTATATE PET/CT parameters (*n* = 30)**	
SSTR-TV	25.02 ± 5.6
SSTR-TL	327 ± 73.1
SUVmax	22.3 ± 2.08
SUVmean	13.18 ± 6.07
**^18^F-FDG PET/CT parameters (*n* = 11)**	
SUVmax	4.44 ± 2.38
SUVmean	2.64 ± 1.45
MTV	16.6 ± 9.54
TLG	58.4 ± 40.8
**Histopathological scores**	
PASS	5 (2–6)
GAPP score	4.5 (2–6)

Data are presented as mean ± SD, median (IQR), or *n* (%), as appropriate. Abbreviations: CT, computed tomography; HU, Hounsfield unit; IQR, interquartile range; SD, standard deviation; Ki-67, Ki-67 proliferation index; SUVmax, maximum standardized uptake value; SUVmean, mean standardized uptake value; SSTR-TV, somatostatin receptor tumor volume; SSTR-TL, somatostatin receptor total lesion activity; MTV, metabolic tumor volume; TLG, total lesion glycolysis; PASS, Pheochromocytoma of the Adrenal Gland Scaled Score; GAPP, Grading System for Adrenal Pheochromocytoma and Paraganglioma.

**Table 2:** Distribution of PASS and GAPP categories in the overall cohort and dual-tracer subgroup.

Cohort	Total, *n*	PASS < 4, *n* (%)	PASS ≥ 4, *n* (%)	GAPP 0–2, *n* (%)	GAPP ≥ 3, *n* (%)
Overall cohort	30	10 (33.3)	20 (66.7)	10 (33.3)	20 (66.7)
Dual-tracer subgroup	11	4 (36.4)	7 (63.6)	4 (36.4)	7 (63.6)

Abbreviations: PASS, Pheochromocytoma of the Adrenal Gland Scaled Score; GAPP, Grading System for Adrenal Pheochromocytoma and Paraganglioma.

**Table 3:** Correlations of biochemical markers and tumor size with PET/CT parameters.

Variable	SSTR-TV (*n* = 30)	SSTR-TL (*n* = 30)	MTV (*n* = 11)	TLG (*n* = 11)
Plasma metanephrine	0.306 (0.100)	0.208 (0.270)	0.146 (0.668)	0.117 (0.732)
Plasma normetanephrine	0.532 (0.002)	0.486 (0.006)	−0.022 (0.949)	−0.025 (0.942)
Urinary metanephrine	0.245 (0.192)	0.147 (0.438)	0.153 (0.653)	0.127 (0.710)
Urinary normetanephrine	0.231 (0.219)	0.182 (0.336)	0.064 (0.852)	0.069 (0.840)
Tumor size	0.725 (<0.001)	0.755 (<0.001)	0.071 (0.836)	0.027 (0.937)

Abbreviations: SSTR-TV, somatostatin receptor tumor volume; SSTR-TL, somatostatin receptor total lesion activity; MTV, metabolic tumor volume; TLG, total lesion glycolysis.

**Table 4:** Correlations of PASS and GAPP Scores with PET/CT Parameters.

Variable (*n*)	Spearman’s ρ	*p* Value
PASS vs. ^18^F SUVmax (*n* = 11)	0.616	0.044
GAPP score vs. ^18^F SUVmax (*n* = 11)	0.720	0.012
PASS vs. ^68^Ga SUVmax (*n* = 30)	0.162	0.392
GAPP score vs. ^68^Ga SUVmax (*n* = 30)	0.062	0.745

Abbreviations: PASS, Pheochromocytoma of the Adrenal Gland Scaled Score; GAPP, Grading System for Adrenal Pheochromocytoma and Paraganglioma; ^18^F, ^18^F-FDG PET/CT; ^68^Ga, ^68^Ga-DOTATATE PET/CT.

**Table 5:** Correlation between ^18^F-to-^68^Ga SUVmax ratio and histopathological scores.

Variable (*n* = 11)	Spearman’s ρ	*p* Value
PASS	0.737	0.010
GAPP score	0.661	0.027

**Table 6:** Patient distribution by SUVmax ratio cutoffs and PASS/GAPP categories.

Histopathological Score Category	Ratio ≥ Cutoff, *n*	Ratio < Cutoff, *n*	Total, *n*
PASS ≥ 4	7	0	7
PASS < 4	1	3	4
GAPP ≥ 3	4	3	7
GAPP 0–2	0	4	4

Values are presented as patient counts. The ^18^F/^68^Ga SUVmax ratio cutoffs were 0.156 for PASS and 0.242 for GAPP. Abbreviations: PASS, Pheochromocytoma of the Adrenal Gland Scaled Score; GAPP, Grading System for Adrenal Pheochromocytoma and Paraganglioma.

## Data Availability

The data presented in this study are available on request from the corresponding author. The data are not publicly available due to privacy and ethical restrictions.
